# Overcoming barriers to the prescription/dispensation of take-home naloxone among French primary care professionals: a regional training initiative

**DOI:** 10.1093/eurpub/ckag074

**Published:** 2026-05-09

**Authors:** Aurélie Aquizerate, Mélanie Duval, Morgane Rousselet, Emmanuelle Jaulin, Emmanuelle Kuhn, Anne-Claire Oger, Isabelle Nicolleau, Solen Pele, Thomas Herault, Pascal Artarit, Edouard-Jules Laforgue, Caroline Victorri-Vigneau

**Affiliations:** Nantes Université, CHU Nantes, Centre d’Evaluation et d’Information sur la Pharmacodépendance-Addictovigilance (CEIP-A), Service de Pharmacologie Clinique, Nantes, France; Nantes Université, CHU Nantes, Centre d’Evaluation et d’Information sur la Pharmacodépendance-Addictovigilance (CEIP-A), Service de Pharmacologie Clinique, Nantes, France; Nantes Université, CHU Nantes, Centre d’Evaluation et d’Information sur la Pharmacodépendance-Addictovigilance (CEIP-A), Service de Pharmacologie Clinique, Nantes, France; SPHERE, Nantes Université, Univ Tours, CHU Nantes, CHU Tours, INSERM, MethodS in Patients-centered outcomes and HEalth Research, Nantes, France; Nantes Université, CHU Nantes, Centre d’Evaluation et d’Information sur la Pharmacodépendance-Addictovigilance (CEIP-A), Service de Pharmacologie Clinique, Nantes, France; UIC22, Service Douleur Soins Palliatifs et Soins de Support, Nantes Université, CHU Nantes, Nantes, France; Réseau Douleur de l‘Ouest (REDO), Nantes, France; Union Régionale des Professionnels de Santé Libéraux (URPS) Pharmaciens Pays de la Loire, Nantes, France; Conseil Régional de l’Ordre des Pharmaciens (CROP) Pays de la Loire, Nantes, France; Structure Régionale d’Appui et d’Expertise (SRAE) en Addictologie des Pays de la Loire, Nantes, France; Union Régionale des Médecins Libéraux (URML) Pays de la Loire, Saint-Sébastien-sur-Loire, France; Direction régionale du Service médical (DRSM) des Pays de la Loire, Nantes, France; Nantes Université, CHU Nantes, Centre d’Evaluation et d’Information sur la Pharmacodépendance-Addictovigilance (CEIP-A), Service de Pharmacologie Clinique, Nantes, France; SPHERE, Nantes Université, Univ Tours, CHU Nantes, CHU Tours, INSERM, MethodS in Patients-centered outcomes and HEalth Research, Nantes, France; Nantes Université, CHU Nantes, Centre d’Evaluation et d’Information sur la Pharmacodépendance-Addictovigilance (CEIP-A), Service de Pharmacologie Clinique, Nantes, France; SPHERE, Nantes Université, Univ Tours, CHU Nantes, CHU Tours, INSERM, MethodS in Patients-centered outcomes and HEalth Research, Nantes, France

## Abstract

Opioid-related mortality is a global issue, and France shows high opioid use disorder indicators. Despite recommendations for wider take-home-naloxone distribution, access remains limited in France, and many primary care professionals lack training. The SINFONI project aims to enhance naloxone education for these providers. A motion-design training program was created and sent to general practitioners (GPs) and pharmacists. A total of 138 GPs and 207 pharmacists participated in the knowledge assessment before and after training. Post-training, significant improvements were observed, and the greatest knowledge gains were in understanding naloxone’s safety (*P* < .001). Our findings primarily identified critical “red flags” that may hinder prescription and dispensing practices.

## Introduction

Opioid overdose deaths are a major public health issue worldwide, particularly in regions affected by synthetic opioids such as fentanyl, and many studies showed the benefits of take-home naloxone (THN) for opioid users [1]. Although France’s situation appears less severe, with an estimated 3.2 opioid overdose deaths per million inhabitants in 2015, recent research using the WHO’s addictovigilance database placed France among the six countries with the highest indicators of opioid use disorder [2]. In France, opioid overdoses involve mainly methadone among drug users and tramadol among patients using analgesics for medical purposes [3]. In March 2022, the French Health Authority (HAS) recommended systematically assessing the appropriateness of prescribing THN to all opioid users and identified eight high-risk situations, including patients with reduced opioid tolerance, those starting or stopping opioid substitution treatment, and individuals with a history of opioid use disorder [4].

In France, GPs and community pharmacists are key actors in the care of people using opioids across all settings (annually, nearly 10 million French individuals receive opioid analgesics, and 160 000 receive opioid agonist treatment). Yet, they remain largely untrained in THN, with no dedicated educational programs available. In France, THN is still mainly limited to specialist addiction services, whereas in countries such as the United States, other channels are used, including retail pharmacies, with over 2.1 million kits dispensed from US pharmacies in 2023 [5]. Thus, a preliminary assessment of these professionals’ practices was conducted in the French Pays de la Loire (PDL) region, including 167 GPs and 158 pharmacists. While almost all managed opioid patients, 47.7% of GPs and 27.8% of pharmacists faced challenges addressing overdose risks during opioid care. However, only 11.5% of GPs and 5.1% of pharmacists prescribed or dispensed THN in the past year. Barriers included a lack of knowledge about naloxone and difficulties understanding overdose risk situations as described by the HAS [6].

The SINFONI project, spearheaded by the Center for Evaluation and Information on Drug Dependence-Addictovigilance of the PDL Region (PDL CEIP-A) with support from the PDL Regional Health Agency (ARS), aims to improve training for primary healthcare professionals. It seeks to address a crucial gap in naloxone education by creating a specialized training tool tailored to their needs.

## Methods

A steering committee was established, comprising representatives from the project’s regional institutional partners. These entities were involved in every phase of the project, from the development of survey and training materials to their dissemination, acting as key channels for reaching 6620 healthcare professionals in the region, including 3730 GPs and 2790 pharmacists. A motion-design training program was specifically created, featuring two videos: one on naloxone’s pharmacodynamic and pharmacokinetic properties and usage guidelines, and another on eight clinical overdose risk situations and patient/family guidance. To assess the impact of this training, an evaluation of the professionals’ knowledge, covering naloxone pharmacology and advice for patients and their families, was performed through an *ad-hoc* questionnaire administered online immediately before and after the video training, from March to December 2023. We compared accuracy rates using a chi-squared test, with the significance level set at 0.05.

## Results

As of January 2024, the videos had accumulated nearly 1400 views. Among the 373 professionals assessed (138 GPs and 207 pharmacists), overall knowledge accuracy was relatively high at baseline (79% and 76%, respectively) and increased significantly after training (91% and 92%; *P* < .001). Major gaps in safety-related knowledge were observed before training. Among GPs, 47% (*n* = 65) incorrectly believed that naloxone could be diverted from its therapeutic use, a proportion that decreased to 7% (*n* = 10) after training. In addition, only 32% (*n* = 44) were aware that naloxone has no pharmacological effects in the absence of opioid agonists, compared with 91% (*n* = 126) after training. Similar gaps were observed among pharmacists: before training, 28% (*n* = 57) considered naloxone diversion possible and 57% (*n* = 118) knew that naloxone is pharmacologically inactive without opioid agonists; these proportions improved to 6% (*n* = 12) and 97% (*n* = 200), respectively, after training.

## Discussion

The SINFONI project is, to our knowledge, the first initiative in France focused on educating primary care professionals about THN, extending its reach beyond addiction medicine and specialized drug treatment centers through the development of a targeted training tool. Although a lack of knowledge about THN was identified by healthcare professionals as a major barrier in prior studies [6, 7], their understanding of naloxone’s pharmacology and pharmacokinetics assessed in our study was actually quite solid, with over 75% accuracy. Our findings primarily identified critical “red flags”—especially misconceptions about naloxone’s safety and potential misuse—that may hinder prescription and dispensing practices. To our knowledge, this specific pattern has not been widely reported, as prescribers’ concerns in the literature more commonly focus on fears that access to THN might paradoxically encourage riskier opioid use through a perceived sense of safety or that excessive naloxone dosing could lead to over-antagonism [8]. In contrast, our results highlight more fundamental knowledge gaps related to naloxone’s pharmacological safety and non-diversion risk. It is therefore particularly noteworthy that the impact assessment in our study demonstrated a significant improvement in understanding, especially concerning naloxone’s safety profile. Furthermore, our training effectively translated the HAS recommendations into clinical practice, enhancing the identification of patients at risk of overdose.

The method employed in our study, which ensured full anonymization, facilitated the dissemination of the training tool to several thousand healthcare professionals in the region. However, it had significant limitations: targeted follow-up was not possible, linking pre- and post-training data was unfeasible, and conducting paired tests could not be achieved. Consequently, this approach does not allow for a thorough assessment of the training’s impact on healthcare professionals’ practices. However, previous studies have demonstrated that a brief curricular intervention covering both THN and the identification of patients at risk of overdose not only enhances physicians’ knowledge but also significantly increases the prescribing and dispensing rates of THN [9].

Finally, one might question whether this training should be limited to healthcare professionals. Indeed, a recent study emphasized the critical need to inform patients and users about the availability of THN and the risk situations of opioid overdose and highlights the importance of broadening the scope of communication beyond just professionals [10]. However, by focusing our efforts on educating healthcare professionals, we empower them to play a key role in transmitting this information directly to their patients, ensuring that the message reaches those most at risk. It is also crucial to recognize the financial barriers that may prevent patients from accessing THN. Like emergency contraception in France, THN should be made available to all without any upfront costs, as recommended by the HAS, to ensure equitable access for everyone. This training also facilitates a systematic evaluation of the appropriateness of providing THN to all opioid users, regardless of their context. Such an approach has the potential to reduce the stigma surrounding addiction, create opportunities to review opioid usage, and promote collaborative assessments of overdose risks between healthcare professionals and opioid users.

Key points

- Opioid-related mortality is a global public health issue, and France shows high indicators of opioid use disorder.
- Enhancing naloxone education is crucial to reduce opioid-related deaths in France.
- Access to naloxone in France remains limited, with insufficient training among healthcare professionals.
- The SINFONI project provides targeted naloxone education for general practitioners and pharmacists using motion design materials.
- Knowledge significantly improved following the intervention, particularly regarding naloxone safety.

Conflict of interest: The authors have no conflicts of interest to declare.

## Data Availability

The data underlying this article will be shared on reasonable request to the corresponding author.
